# 2,5-Hexanedione mediates neuronal apoptosis through suppression of NGF via PI3K/Akt signaling in the rat sciatic nerve

**DOI:** 10.1042/BSR20181122

**Published:** 2019-02-12

**Authors:** Enjun Zuo, Cong Zhang, Jun Mao, Chenxue Gao, Shuhai Hu, Xiaoxia Shi, Fengyuan Piao

**Affiliations:** 1 College of Stomatology, Dalian Medical University, Dalian 116044, China; 2 Department of Food Nutrition and Safety, Dalian Medical University, Dalian 116044, China; 3 Department of Pathology, Dalian Medical University, Dalian 116044, China; 4 Department of Occupational and Environmental Health, Dalian Medical University, Dalian 116044, China

**Keywords:** 2,5-hexanedione, apoptosis, NGF, PI3K/Akt signaling pathway, sciatic nerve

## Abstract

Because precise mechanism for 2,5-hexanedione (HD)-induced neuronal apoptosis largely remains unknown, we explored the potential mechanisms both *in vivo* and *in vitro*. Rats were intraperitoneally exposed to HD at different doses for 5 weeks, following which the expression levels of nerve growth factor (NGF), phosphorylation of Akt and Bad, dimerization of Bad and Bcl-xL, as well as the release of cytochrome *c* and the caspase-3 activity were measured. Moreover, these variables were also examined *in vitro* in HD-exposed VSC4.1 cells with or without a PI3K-specific agonist (IGF-1), and in HD-exposed VSC4.1 cells with or without a PI3K-specific inhibitor (LY294002) in the presence or absence of NGF. The data indicate that, as the concentration of HD increased, rats exhibited progressive gait abnormalities, and enhanced neuronal apoptosis in the rat sciatic nerve, compared with the results observed in the control group. Furthermore, HD significantly down-regulated NGF expression in the rat sciatic nerve. Moreover, suppression of NGF expression inhibited the phosphorylation of Akt and Bad. Meanwhile, an increase in the dimerization of Bad and Bcl-xL in mitochondria resulted in cytochrome *c* release and caspase-3 activation. In contrast, HD-induced apoptosis was eliminated by IGF-1. Additionally, NGF supplementation reversed the decrease in phosphorylation of Akt and Bad, as well as reversing the neuronal apoptosis in HD-exposed VSC4.1 cells. However, LY294002 blocked these effects of NGF. Collectively, our results demonstrate that mitochondrial-dependent apoptosis is induced by HD through NGF suppression via the PI3K/Akt pathway both *in vivo* and *in vitro.*

## Introduction

N-Hexane, a non-polar solvent, is widely regarded as a cheap, easily evaporated, largely unreactive chemical that is used in many industrial processes. Occupational toxicological studies have shown that *n*-hexane is harmful to the nervous system of those who have exposed to it [1–3]. Chronic exposure to *n*-hexane induces peripheral neuropathy in both humans and animals [4–6]. 2,5-Hexanedione (HD), an endogenous metabolite of *n*-hexane, has been identified as a pathogenic agent of *n*-hexane neurotoxicity [7,8]. Public-health is indeed faced with threats of citizen exposure to *n*-hexane and HD more and more regularly. There is growing evidence that HD induces apoptosis in neurons or its derived cell lines. For instance, it has been reported that exposure to HD at a low-concentration induces apoptosis in murine dorsal root ganglion neurons [9,10]. In addition, the level of apoptosis increases in SK-N-SH cells (human neuroblastoma) following exposure to HD [11]. Additionally, our previous study demonstrated that apoptosis increases in the rat spinal cord upon exposure to HD [12] Moreover, a morphological study of neuronal populations indicated that HD causes a marked reduction in cell numbers and an associated reduction in cell size [10]. Taken together, these results suggest that HD induces neuronal apoptosis, which may underscore the basic mechanism for its neurotoxicity. However, identification of the signaling pathways that underlie the potential mechanisms of HD-induced apoptosis in rat sciatic nerve requires further investigation.

An increasing number of reports have indicated that the phosphatidylinositol3-kinase (PI3K)/Akt signaling pathway is involved in cell survival and apoptosis [13,14]. Activation of PI3K/Akt signaling blocks the mitochondrial apoptotic pathway. Furthermore, inhibition of PI3K/Akt signaling activates the apoptotic pathway [15–17]. The expression and phosphorylation of Akt, through PI3K activation, prevent the apoptosis that is induced by the inhibition of Akt phosphorylation [18–20]. Following Akt phosphorylation, the activation of the PI3K/Akt signaling pathway is mediated by Bcl-2 phosphorylation, followed by subsequent inhibition of Bcl-2 and Bad phosphorylation [21–23]. Phosphorylated Bad then binds to the 14-3-3 protein, releasing Bcl-xL in to the cytoplasm. The release of Bcl-xL, in turn, unblocks the translocation of the Bax protein to the mitochondria, resulting in the disruption of the mitochondrial membrane potential (MMP). The subsequent release of cytochrome *c* (Cyt c) from the mitochondria induces apoptosis [24,25].

Neurotrophins are protein molecules that are secreted by nerve cells. Neurotrophins are involved in neuronal survival [26,27]. These molecules can protect neurons against neurotoxic insults [28,29]. A previous study demonstrated that nerve growth factor (NGF) reduces cell death through the activation of the PI3K/Akt signaling pathway [30]. The same group also showed that NGF attenuates thapsigargin-mediated apoptosis of PC12 cells via PI3K/Akt signaling pathway [31]. Moreover, the expression level of NGF has an important impact on the activity of the PI3K/Akt signaling pathway. NGF withdrawal reduces the activity of PI3K/Akt signaling pathway and induces apoptosis in sympathetic neurons cultured *in vitro* [30]. Additionally, abnormal NGF expression might have an adverse effect on neuronal survival via PI3K/Akt signaling pathway [31].

Other studies have shown that ethanol exposure results in a significant reduction of NGF expression in the hippocampus and cortex in male mice [32]. While NGF expression can also be reduced through chronic exposure to amphetamine in the same areas [33]. These results indicate that NGF is a potential target for the toxic interference mediated by certain neurotoxicants. Based on these observations, we hypothesized that NGF expression is inhibited by HD exposure in the sciatic nerve, and that HD-induced neuronal apoptosis is mediated by NGF inhibition through the suppression of PI3K/Akt signaling.

In our study, we aimed to determine whether regular exposure to HD (100, 200 and 400 mg/kg per day for 5 weeks; i.p.) induces neuronal apoptosis in the rat sciatic nerve. We also assessed the expression level of NGF, the phosphorylation of Akt and Bad, the dimerization of Bad/Bcl-xL, the release of cytochrome *c* as well as caspase-3 activity. In addition, we examined the effect of HD exposure combined with other interventions using a PI3K-specific agonist (IGF-1), and a PI3K/Akt pathway inhibitor (LY294002) on VSC4.1 cells *in vitro*. Our results suggest that HD induces the suppression of endogenous NGF and mitochondrial-dependent apoptosis through down-regulation of PI3K/Akt signaling in the rat sciatic nerve *in vivo*. Our results also suggest that the suppression of endogenous NGF is involved in HD-induced neuronal apoptosis through down-regulation of PI3K/Akt signaling *in vitro*. Our aims were to determine the specific effects of HD exposure on neuronal apoptosis and to evaluate potential signaling pathways that may be involved in regulation of HD-induced apoptosis both *in vivo* and *in vitro*.

## Materials and methods

### Animal models

Male adult SD rats weighing 200–230 g were raised in Animal Facilities of Dalian Medical University in accordance with the Guidelines for Experimental Animal Use in Dalian Medical University. Animals were randomly divided into four groups (*n* = 10 per group) after a 7-day acclimatization period. As previously reported, HD was diluted in normal saline. Rats in the experimental groups were administrated with HD solution (i.p.) at a dosage of 100, 200 or 400 mg/kg at a volume of 3 ml/kg per day and five times per week. The rats in the control group were treated with the same amount of normal saline. During the experiment, the gait abnormalities and health status of these rats were observed and recorded daily. Rats were killed by cervical decapitation after 5 weeks of HD or saline administration. The sciatic nerves were dissected out and frozen in liquid nitrogen as soon as possible after killing, before being stored at −80°C. All of the procedures using the animal model were performed following the guidelines from the Animal Ethics Committee of the Dalian Medical University.

### Assessment of neurological defects

2,5-HD-induced neurological defects were assessed using a gait abnormality rating scale, which is widely accepted as a reliable and sensitive rating scale of neurological defects caused by toxicants. Regarding the measurement of gait abnormalities, the rats were kept in a transparent box made out of plexiglass for 3-min successive observation [4,34]. The defects were rated accordingly, based on the extent of the gait abnormality as follows: a score of 1 represents a normal, unaffected gait, while scores of 2, 3 and 4 represent a slightly affected gait, a moderately affected gait and a severely affected gait, respectively. Double-blinded experiments were performed by a well-trained observer who was not involved in any previous animal care or 2,5-HD exposure, and hence was assigned for behavioral evaluation. Three successive measurements were averaged for each 2,5-HD-exposed or control rats [35].

### VSC4.1 motor neuron cell culture

Motor neurons that originated from the Ventral sciatic nerve 4.1 (VSC4.1) [35,37,38] were produced and cultured. Briefly, embryonic motor neurons from the rat ventral sciatic nerve were fused with mouse neuroblastoma cells (N18TG2), which were grown at 37°C in poly-L−ornithine coated flasks in a 5% CO_2_ incubation system. The culture media for these cells were composed of a mixture of 10 ml DMEM (Sigma, St. Louis, U.S.A.), 15 mM HEPES (Sigma, St. Louis, U.S.A.), 1% (Sigma, St. Louis, U.S.A.), 1.5 g/l NaHCO_3_ (Sigma, St. Louis, U.S.A.), 2% Sato’s components (Beyotime, Shanghai, China), 1% penicillin and streptomycin (Beyotime, Shanghai, China) and 15% heat-inactivated fetal bovine serum (Hyclone, Logan, UT, U.S.A.).

### HD exposure in VSC4.1 cells

To determine the effect of HD on the NGF expression *in vitro*, VSC4.1 cells were divided into three groups, including one control group (normal medium) and two experimental groups (treated with 5 or 10 mM HD), and the expression level of NGF was examined.

To determine whether HD induces apoptosis via PI3K/Akt signaling pathway, VSC4.1 cells were cultured with DMEM containing 2% FBS for 24 h. The cells in each group were then exposed to 10 mM HD, 10 mM 2,5-HD plus 50 ng/ml IGF-1 or DMEM medium without HD, respectively. Apoptosis markers including Bad, Bcl-xL, cytochrome *c*, p-Bad, and markers for PI3K/Akt signaling pathway including Akt and p-Akt, were examined.

To determine whether the effect of NGF on HD-induced apoptosis was mediated by the PI3K/Akt signaling pathway, VSC4.1 cells were cultured with DMEM containing 2% FBS for 24 h. The cells in each group were then exposed to 10 mM HD, 10 mM HD plus 50 μg/l NGF, 10 mM HD plus 50 μg/l NGF and 25 μM LY294002, or DMEM medium without HD, respectively. Apoptosis markers including Bad, Bcl-xL, cytochrome *c*, p-Bad and PI3K/Akt signaling pathway markers including Akt and p-Akt, were then examined.

### TUNEL assay

An *In Situ* Cell Death Detection Kit (Roche, Mannheim, Germany) was used for both VSC4.1 cells and 5-µm sections of rat sciatic nerve. The TUNEL-positive neurons were subsequently detected using a fluorescence microscope (×400 magnification). Six fields in each well or each section were randomly selected, and the apoptosis index (AI) was calculated using the following equation: AI = (number of TUNEL-positive neurons / total neuron number [DAPI]) × 100%.

### Immunofluorescence

VSC4.1 cells were fixed through incubation with a 4% paraformaldehyde solution for 10 min, followed by 0.3% TritonX-100 solution for 5 min and 10% goat serum albumin blocking solution at room temperature for 1 h. After the appropriate preparations, the cells were incubated with the primary antibody against Cyt c (1:500, Abcam, Cambridge, U.K.) at 4°C overnight, followed by an incubation with the secondary antibody labeled with Alexa Fluor 488 (Jackson, West Grove, U.S.A.) at room temperature for 45 min. Before mounting, cells were incubated for 5-min in 5 μg/ml DAPI solution. All images were captured using an Olympus DP72 fluorescence microscope (Olympus, Japan) with a mounted camera. The objective that was used for all image acquisition was selected to detect Cyt c release in VSC4.1 cells. Excitation and emission wavelengths for Cyt c were 488/525 nm, and 360/460 nm for DAPI. The sections were covered by glycerine, and the magnification is ×100 or ×200*.*

### Isolation of mitochondrial and cytosol protein

A tissue Mitochondria Isolation Kit (Beyotime, Shanghai, China) was used to specifically isolate the mitochondria from the rat sciatic nerve. The sciatic nerve was homogenized in Reagent A of the kit at a ratio of 10 µl/mg (tissue weight). The mixture was centrifuged (600 ***g***) for 5 min, an additional centrifugation (11000 ***g***) was repeated on the supernatant obtained for another 10 min at 4°C. The pellets were resuspended and were centrifuged again according to above procedures. The cytosol fraction (supernatant) was finally collected and transferred to a new preservation tube.

### Co-immunoprecipitation

Approximately 100 μg of the mitochondrial fractions that were isolated from VSC4.1 cells or the rat sciatic nerves were used for co-immunoprecipitation (Co-IP). A mixture of protein A sepharose and protein G sepharose (protein A + G sepharose; 20 μl; Beyotime, Shanghai, China) was added to these samples for a 30-min incubation at 4°C. Following incubation, the mixture was centrifuged (12000 ***g***) for 10 min. The supernatant that was obtained was then incubated with the primary antibody against Bcl-xL (2 μg; 1:1000) and 15 μl of protein A + G sepharose (50% slurry) for 5 h at 4°C. Another round of centrifugation (12000 ***g***) was then performed for 1 min and the supernatant was collected and transferred to a new preservation tube for Western blot analysis.

### Western blot

Samples were homogenized using ice-cold RIPA Lysis Buffer (Beyotime, Shanghai, China) supplemented with 1% proteinase inhibitor cocktail (100×). The proteins examined (20 µg/lane) were separated by SDS-PAGE and then electrotransferred onto the polyvinylidene fluoride (PVDF) membrane (Millipore, Darmstadt, Germany). The membrane was then incubated at 4°C overnight with primary antibodies for NGF (1:500, Cell Signaling Technology, Danvers, U.S.A.), Akt (1:1000, Sigma, St. Louis, U.S.A.), p-Akt (ser-473) (1:1000, Sigma, St. Louis, U.S.A.), Bad (1:1000, Sigma, St. Louis, U.S.A.), p-Bad (ser-136) (1:1000, Sigma, St. Louis, U.S.A.), cytochrome c polyclonal (1:100, CST, Mass, U.S.A.), VDAC (1:1000, CST, Mass, U.S.A.) and β-actin (1:500, ZS-Bio, Shanghai, China). Immunoreactivity was measured using second antibodies conjugated with horseradish peroxidase (1:5000, Sigma, St. Louis, U.S.A.) through a luminol-enhanced chemoluminescence assay (ECL; Beyotime, Shanghai, China), which was visualized and quantified by the ChemiDoc™ XRS+ System (Bio-rad, Richmond, U.S.A.) or the UVP BioSpectrum Multispectral Imaging System (Ultra-Violet Products Ltd. Upland, U.S.A.). Expression levels of Cyt c as well as other apoptosis-related proteins in the mitochondria and/or cytoplasm were measured, while β-actin/VDAC were regarded as the loading controls to quantify the relative expression of protein in cytosol and mitochondrial, respectively.

### Detection of caspase-3 activity

The lysis buffer supplied with the caspase-3 activity kit (Beyotime, Shanghai, China) was used to isolate caspase-3 from VSC4.1 cells or rat sciatic nerve. The assay of caspase-3 activity was measured in a 96-well plate by adding 40 µl lysis sample to 50 µl reaction buffer containing 1% NP-40, 20 mM Tris-HCl (pH 7.5), 137 mM NAD and 10% glycerol, which was supplemented with 2 mM caspase-3 substrate (Ac-DEVD-pNA; 10 µl) at 37°C for 2 h. The result of each specific example was obtained by measuring the absorbance at 405 nm in a microplate reader.

### Statistical analysis

All statistical analyses were carried out using SPSS 13.0 statistical software. The results are expressed as mean ± standard deviation (S.D.). The results from different groups were compared using one-way ANOVA, followed by LSD or Dunnett’s multiple comparison tests. A *P*-value less than 0.05 was accepted as being statistically significant. All experiments involving cell detection were performed in triplicate, and all tissue assays were repeated for three times.

## Results

### Behavioral assessment of rats exposed to HD

**Figure 1:**
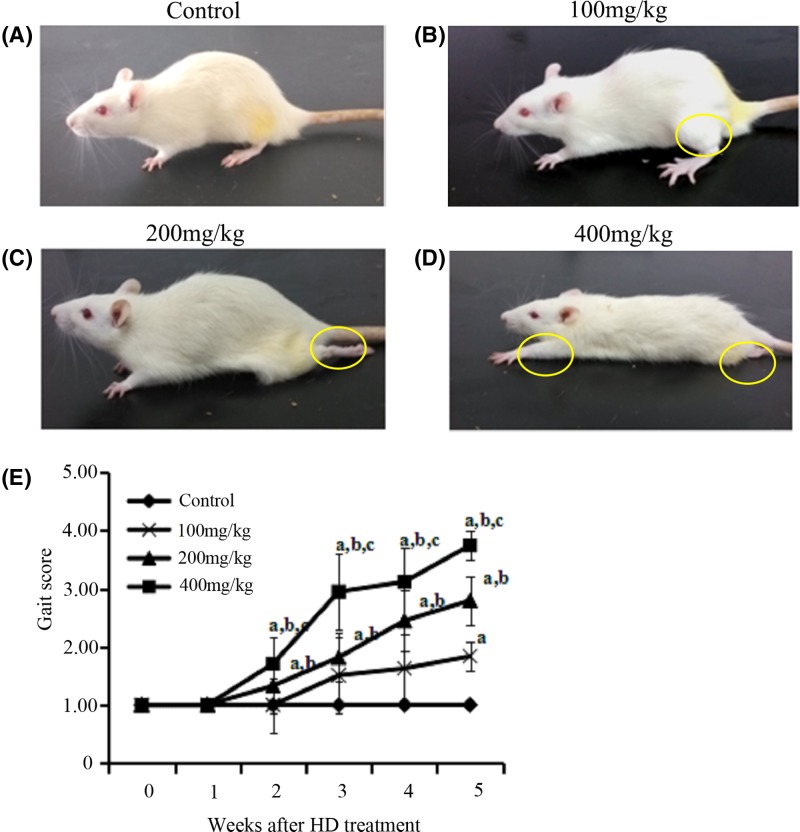
Behavioral assessment of animal model exposed to HD (**A**) Rat in control group. (**B**) Rat in group exposed to 100 mg/kg HD. (**C**) Rat in group exposed to 200 mg/kg HD. (**D**) Rat in group exposed to 400 mg/kg HD. (**E**) Gait scores of rats in all groups. ^a^*P*<0.05, compared with the control group results; ^b^*P*<0.05, compared with the results of the group that was exposed to 100 mg/kg HD; ^c^*P*<0.05, compared with the results of the group that was exposed to 200 mg/kg HD.

The results of neurobehavioral observations in four groups of rats are shown in Figure 1A–D. No abnormal behavioral phenotypes were observed in the control group during the experiment. Conversely, administration of 400 mg/kg HD treatment for 5 weeks caused a severely abnormal gait (dragging hind limbs, inability to rear and even paralysis of limbs) in these rats. Administration of 200 mg/kg HD treatment for 5 weeks caused a similar but less severe phenotype in rats. The rats that were exposed to 100 mg/kg HD for 5 weeks exhibited only tip-toe walking, slight ataxia and foot splay. Moreover, as shown in Figure 1E, the gait scores of the rats in the experimental groups gradually increased from the second week after HD administration and were dose-dependently elevated. At the end of the experiment, the average gait score value of the rats was 1.00 in the control group, 1.84 in the group treated with 100 mg/kg HD, 2.80 in the group treated with 200 mg/kg HD and 3.75 in the group treated with 400 mg/kg HD, respectively. There were significant differences in the gait score between the experimental groups and the control group (*P*<0.05), revealing that HD induced neurological defects in rats.

### The effect of HD on mitochondrial-dependent apoptosis in the rat sciatic nerve

**Figure 2:**
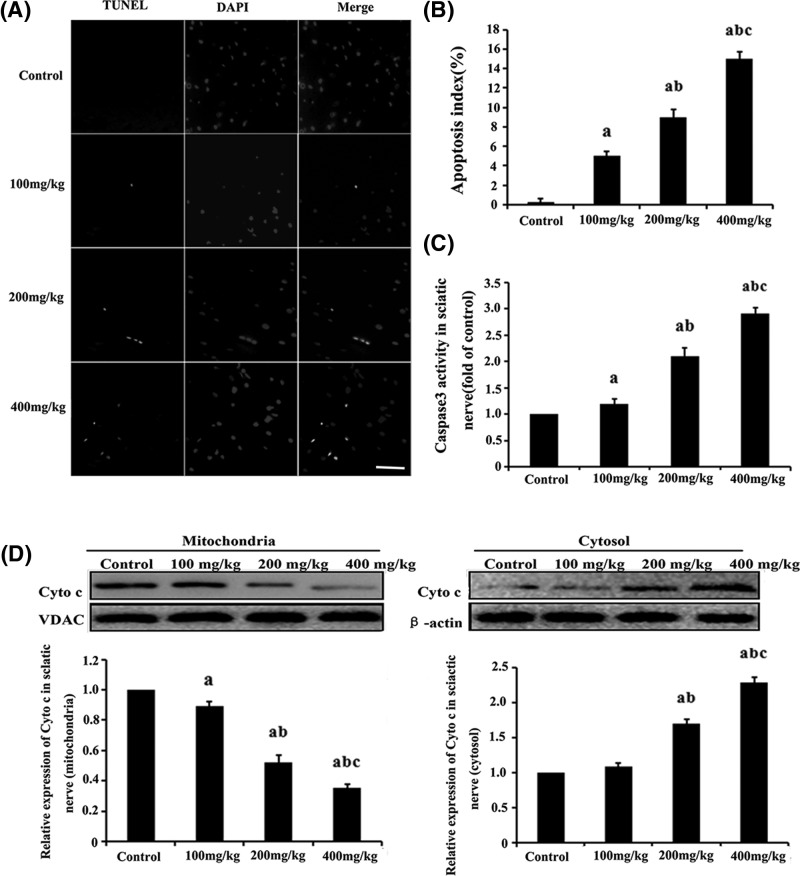
The effect of HD on the apoptosis induction test in the rat sciatic nerve (**A**) TUNEL labeling with DAPI counter staining to detect apoptotic cells in the rat sciatic nerve (scale bar = 20 μm). (**B**) Quantification of TUNEL-positive cells. (**C**) Detection of caspase-3 activity in the rat sciatic nerve. (**D**) The expression level of Cyt c determined by Western blot analysis in the mitochondria or cytosolic fractions and their quantifications. ^a^*P*<0.05, compared with the control group results; ^b^*P*<0.05, compared with the results of the group exposed to 100 mg/kg HD; ^c^*P*<0.05, compared with the results of the group exposed to 200 mg/kg HD.

Apoptotic neurons in rat sciatic nerve were detected as TUNEL-positive cells (Figure 2A). The number of TUNEL-positive cells in the sciatic nerve of HD-exposed rats was higher than that of the control group. Moreover, the apoptosis index was 5.1 ± 0.38%, 9.2 ± 0.51% and 12.8 ± 0.47% in the groups exposed to 100, 200 and 400 mg/kg of HD, respectively. These values were significantly higher than that of the control group (0.1 ± 0.21%; *P*<0.05; Figure 2B). However, almost no TUNEL-positive cells were detected in the control group.

Mitochondrial apoptosis, which plays a pivotal role in neuronal survival, is associated with the release of Cyt c from the mitochondria and caspase-3 activation. Our results show that the expression level of Cyt c in the mitochondria was significantly lower in the groups that were exposed to HD than those observed in the control group. Meanwhile, the Cyt c expression level obtained from the cytosolic fraction was markedly higher in the rat sciatic nerve that was exposed to HD than in the control group. Accordingly, Cyt c expression level was reduced by approximately 64% of the control value in the group that was treated with 400 mg/kg HD (Figure 2D). HD-induced neurotoxicity also increased caspase-3 activity in the rat sciatic nerve, and the activity was increased by approximately 125% in the group that was treated with 400 mg/kg HD (Figure 2C).

### The effect of HD on the expression of Akt, p-Akt, Bad and p-Bad in the rat sciatic nerve

**Figure 3:**
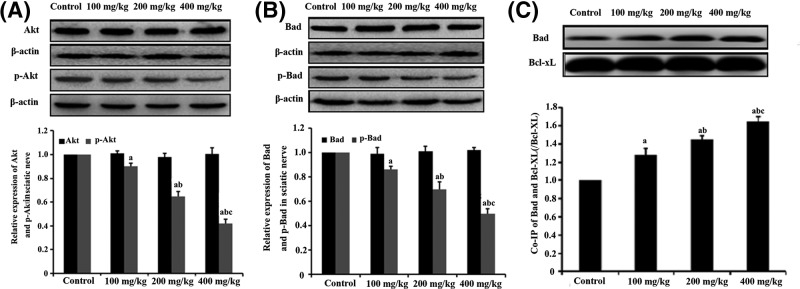
The effect of HD on apoptotic gene expression and phosphorylation as revealed by the expression of Akt, p-Akt, Bad and p-Bad in the rat sciatic nerve (**A**) The expression levels of Akt and p-Akt in the sciatic nerve of HD-exposed rats, as displayed by Western blot and quantification. (**B**) The expression levels of Bad and p-Bad in the sciatic nerve of HD-exposed rats, as displayed by Western blot and quantification. (**C**) Detection of dimerization level of Bad/Bcl-xl from the mitochondria of the rat sciatic nerve tissue using Co-IP. ^a^*P*<0.05, compared with control group results; ^b^*P*<0.05, compared with the results of the group exposed to 100 mg/kg HD; ^c^*P*<0.05, compared with the results of the group exposed to 200 mg/kg HD.

The expression levels of apoptotic genes, such as Akt, p-Akt, Bad and p-Bad, were tested by Western blot analysis (Figure 3A,B). The expression levels of Akt and Bad in the rat sciatic nerve tissue were not significantly different between the HD-exposed groups and the control group (*P*>0.05). The expression levels of p-Akt and p-Bad, however, were significantly lower in rats that was exposed to HD than in the control group (*P*<0.05). Accordingly, the expression levels of p-Akt and p-Bad were reduced by 55% and 50%, respectively, in the group that was treated with 400 mg/kg HD compared with the results in the control group. This effect was considered to be dose-dependent.

To evaluate the protein interaction in the mitochondrial fraction of the sciatic nerve, Co-IP of Bad with Bcl-Xl, which is regarded as an anti-apoptotic protein, was performed (Figure 3C). Bcl-Xl (IP: Bcl-xL) and BAD (WB: Bad) in the lysate were measured by Western blot analysis as an internal control before Co-IP, respectively. The dimerization of Bad and Bcl-xL was significantly higher in the mitochondrial fraction of the rats from the experimental groups than in the control group. Accordingly, dimerization of Bad and Bcl-xL was increased by approximately 65% in the group that was treated with 400 mg/kg HD compared with the control values.

### The effects of IGF-1 on HD-induced mitochondrial apoptosis via the PI3K/Akt/Bad signaling pathway

**Figure 4:**
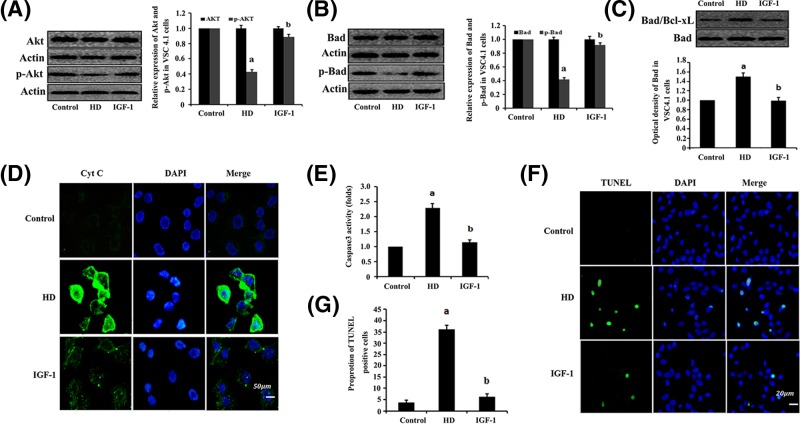
IGF-1 inhibition of HD-induced mitochondrial apoptosis via the PI3K/Akt signaling pathway in the VSC4.1 cells VSC4.1 cells were exposed to 10 mM 2,5-HD, 10 mM 2,5-HD + 50 ng/ml IGF-1 for 24 h. Western blot analysis was used to detect Akt, p-Akt, Bad and p-Bad protein expression. (**A** and **B**) Detection of expression levels of Akt and p-Akt (A) or Bad and p-Bad (B), respectively. Data are presented as mean ± S.D. (*n*=3). (**C**) Examination of the dimerization of Bad/Bcl-xL complex in the mitochondria using Co-IP. (**D**) Immunofluorescence test for Cyt c release in VSC4.1 cells. (**E**) The detection of caspase-3 activity. (**F**) TUNEL labeling with DAPI counter staining was performed to detect apoptotic cells in VSC4.1 cells. (**G**) Quantification of the abundance ratio of TUNEL-positive cells in each condition. ^a^*P*<0.05, compared with control group results; ^b^*P*<0.05, compared with the results of the group exposed to 10 mM 2,5-HD.

To determine whether HD-induced mitochondrial apoptosis via the PI3K/Akt/Bad signaling pathway, effects of IGF-1 (a PI3K-specific agonist) on the expression levels of Akt, p-Akt, Bad and p-Bad proteins were tested in VSC4.1 cells exposed to HD (Figure 4A,B). Moreover, apoptosis, caspase-3 activity and Cyt c expression were also examined in the VSC4.1 cells, respectively (Figure 4D–G). The results showed that HD significantly down-regulated the expression levels of p-Akt and p-Bad, and decreased the dimerization level of Bad with Bcl-xL in the mitochondria (Figure 4C) compared with the levels that were observed in the VSC4.1 cells control group (*P*<0.05). The apoptosis, caspase-3 activity and Cyt c expression were markedly higher in the VSC4.1 cells that were exposed to HD than in the control group. Moreover, this particular drug-induced apoptosis in VSC4.1 cells, which was induced by HD, was rescued by IGF-1 administration. Accordingly, the expression levels of p-Akt and p-Bad increased by approximately 114% and 125%, respectively, compared with the control values, in the group treated with 10 mM 2,5-HD + 50 ng/ml IGF-1; while the dimerization levels of Bad with Bcl-Xl, caspase-3 activity and number of apoptotic cells were reduced by approximately 50%, 45% and 86%, respectively, compared with the results in the group that was treated with 10 mM 2,5-HD.

### The effect of HD on NGF in the rat sciatic nerve tissue and VSC4.1 cells

**Figure 5:**
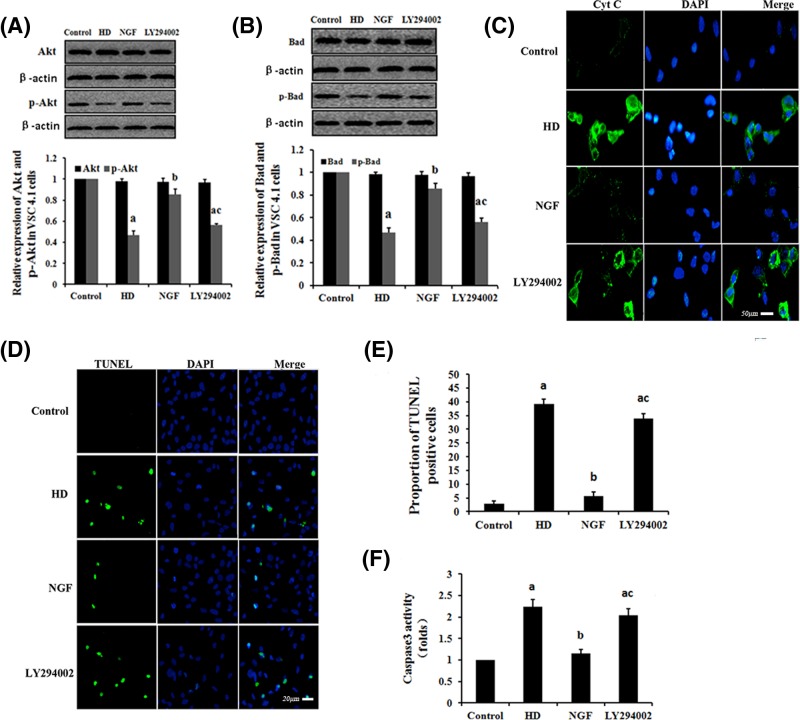
The effect of NGF on HD-induced mitochondrial apoptosis via the PI3K/Akt signaling pathway VSC4.1 cells were exposed to 10 mM 2,5-HD, 10 mM 2,5-HD + 50 μg/l NGF or 10 mM 2,5-HD + 50 μg/l NGF + 25 μM LY294002 for 24 h. Western blot analysis was used to detect the Akt, p-Akt, Bad and p-Bad protein expression levels. (**A** and **B**) Detection of expression levels of Akt and p-Akt (A) or Bad and p-Bad (B) in VSC4.1 cells, respectively. Data are presented as mean ± S.D. (*n*=3). (**C**) Cyt c was examined by immunofluorescence test in each condition (scale bar = 50 μm). (**D**) TUNEL labeling with DAPI counter staining was performed to detect apoptotic cells in each condition (scale bar = 20 μm). (**E**) Quantification of the abundance ratio of TUNEL-positive cells in each condition. (**F**) Detection of caspase-3 activity in each condition. ^a^*P*<0.05, compared with the control group results; ^b^*P*<0.05, compared with the results of the group exposed to 10 mM 2,5-HD; ^c^*P*<0.05, compared with the results of the group exposed to 10 mM 2,5-HD + 50 μg/l NGF.

The NGF protein expression in the rat sciatic nerve was analyzed by Western blot analysis (Figure 5A). The NGF protein expression in the rat sciatic nerve was significantly lower in the experimental group than in the control group. In particular, NGF expression in the group that was treated with 400 mg/kg HD reduced by approximately 39% compared to the control (*P*<0.05). *In vitro* study was also performed using VSC4.1 cells. The expression level of NGF protein was significantly lower in the HD-exposed VSC4.1 cells than in the control group. Accordingly, NGF expression in the group treated with 10 mM HD reduced by approximately 62% compared with the control (Figure 5B).

### The effects of NGF on HD-induced mitochondrial-dependent apoptosis via the PI3K/Akt pathway

**Figure 6:**
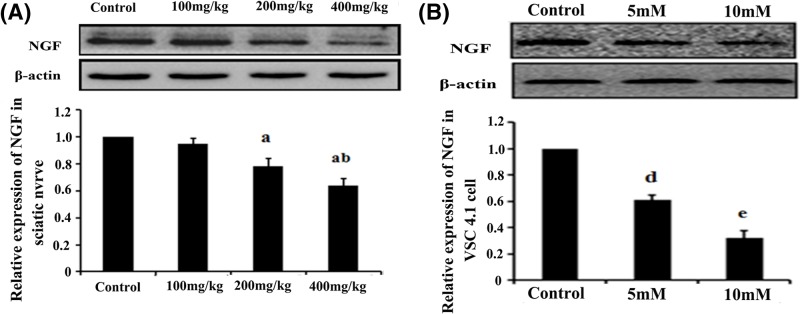
The effect of HD on NGF expression in the rat sciatic nerve and VSC4.1 cells (**A** and **B**) Detection of NGF expression in the rat sciatic nerve (**A**) and VSC4.1 cells (**B**) in each condition. Data are expressed as mean ± S.D. (*n* = 3 in each group); ^a^*P*<0.05, compared with the control group results; ^b^*P*<0.05, compared with the results of the group exposed to 100 mg/kg HD; ^c^*P*<0.05, compared with the results of the group exposed to 200 mg/kg HD; ^d^*P*<0.05, compared with the results of the control group; ^e^*P*<0.05, compared with the results of the group exposed to 5 mM HD.

The expression levels of Akt, p-Akt, Bad and p-Bad proteins in VSC4.1 cells that were exposed to HD were tested by Western blot analysis (Figure 6A,B). The results show that the expression levels of the p-Akt and p-Bad were significantly lower in the HD-exposed cells than in the control group (*P*<0.05). In cells exposed to HD, NGF administration lead to a significant reinduction of p-Akt and p-Bad expression compared with that observed in the HD-exposed cell group (*P*<0.05). The antagonistic effects of NGF were inhibited by the addition of LY294002, which abolished PI3K/Akt signaling by directly inhibiting PI3K. These results indicate that NGF administration activated the PI3K/Akt signaling pathway in the VSC4.1 cells that were exposed to HD. To examine whether NGF antagonizes HD-induced apoptosis via the PI3K/Akt signaling pathway, we investigated the apoptosis, caspase-3 activity and Cyt c expression in VSC4.1 cells, respectively (Figure 6C–F). Apoptosis, caspase-3 activity and Cyt c release were significant higher in in VSC4.1 cells that were exposed to HD than in the control group. However, NGF supplementation attenuated these increases in the HD-exposed VSC4.1 cells. Moreover, the capacity for NGF to attenuate this effect was reversed by LY294002. The expression levels of p-Akt and p-Bad were reduced by approximately 32% and 30%, respectively, compared with the control, in groups that were treated with 10 mM 2,5-HD + 50 μg/l NGF + 25 μM LY29400; while caspase-3 activity and number of apoptotic cells were increased by 75% and 600%, respectively, in comparison with the group treated with 10 mM 2,5-HD + 50 μg/l NGF.

## Discussion

*n*-Hexane, an important non-polar solvent, is widely used in global manufacturing industries. The main natural metabolite of *n*-hexane *in vivo* is HD, which is commonly regarded to be directly involved in the neurotoxicity and neurodegenerative diseases that are mediated by *n*-hexane. It has been well documented that *n*-hexane and HD cause toxic neuropathies and impairments in the central and peripheral nervous systems of humans and animals [4]. In our study, rats that were exposed to a low dose of HD (100 mg/kg) exhibited toe-tip walking and mild ataxia. These symptoms were not observed in the control group. In addition, the rats in the group exposed to a higher dose of HD (400 mg/kg) exhibited symptoms of slaying feet, dragging hind legs and inability to rear. Moreover, the rats in the group exposed to 200 mg/kg HD also showed similar but less severe phenotypes, compared with those rats exposed to 400 mg/kg HD. Taken together, our results indicate that HD is involved in the emergence of neuropathies in the rats.

Neuronal apoptosis plays an important role in the homeostasis of the central and peripheral nervous systems and is a highly regulated process. Extensive disruption of this homeostasis induces apoptosis, e.g. HD treatment [38,39] causes pathological and protective responses in the whole body, and leads to various neurological diseases. In our study, apoptosis in the rat sciatic nerve exposed to HD was tested by TUNEL assays. The number of TUNEL-positive cells in the groups exposed to HD was significantly higher than that observed in the control group. This effect was considered to be dose-dependent. These results indicate that HD administration induced neuronal apoptosis in the rat sciatic nerve, which may then subsequently cause neurological disorders and behavioral deficits in the rats.

The PI3K/Akt signaling pathway is commonly regarded as a pivotal pathway for cell survival [40,41]. Akt plays a major role in the PI3K/Akt signaling pathway. It is widely accepted that Akt phosphorylation inhibits apoptosis. Meanwhile, conversely, apoptosis is induced by the inhibition of Akt phosphorylation [42,43]. In our study, no significant change was detected in the expression level of the Akt protein in rat sciatic nerve between the HD-exposed groups and the control group. However, the expression level of p-Akt in rat sciatic nerve was significantly lower in the group exposed to HD than in the control group. Additionally, *in vitro* results, from experiments using VSC4.1 cells, show that HD exposure down-regulates p-Akt expression, which is consistent with the current *in vivo* results. These results indicate that HD inhibits Akt phosphorylation in the rat sciatic nerve.

Bad is one of the apoptosis-inducing members of the Bcl-2 family, and Akt is the main regulator of the pro-apoptotic activity [44]. Previous studies have shown that phosphorylation of Akt can induce Bad phosphorylation, which inactivates Bad [45,46]. In contrast, inhibition of Akt phosphorylation reduces the expression level of phosphorylated Bad, which in turn activates Bad [47]. Dephosphorylated Bad binds to its anti-apoptotic partner, Bcl-xL, to release Cyt c from the mitochondria, which leads to apoptosis [48,49]. In our study, the expression levels of Bad and p-Bad in rat sciatic nerve were assessed. There was no significant difference in the Bad expression in rat sciatic nerve between the HD-exposed group and the control group. However, Bad phosphorylation was significantly lower in the rats exposed to HD than in the control group. These results indicated that HD suppresses Bad phosphorylation in rat sciatic nerve. Notably, the level of dephosphorylated Bad that was bound to Bcl-xL obtained from the mitochondrial fraction of rat sciatic nerve was higher in the group exposed to HD than in the control group by using co-immunoprecipitation. This result confirmed the specificity of the dimerization of Bad with Bcl-xL in the mitochondria. Furthermore, our results also showed that the level of Cyt c in the cytosol fraction was higher in the rats that were exposed to HD than the control group and the level of Cyt c in the mitochondrial fraction was lower in the rats that were exposed to HD than the control group. Supportive evidence were provided by the *in vitro* experiments, which showed that HD-induced neurotoxicity in VSC4.1 cells was mediated by a significant increase in caspase-3 activity. Interestingly, HD-induced abnormalities and behavioral impairments were rescued by the treatment of a PI3K-specific agonist (IGF-1). These results suggested that HD inhibits the PI3K/Akt signaling pathway, which is involved in HD-induced mitochondrial-dependent apoptosis in rat sciatic nerve.

NGF plays a pivotal role in the regulation of neuronal development, plasticity and disease [50]. Defects in either the expression and modification or the secretion of NGF cause various dysfunctional phenotypes in the nervous system. Our studies indicate that NGF activates the PI3K/Akt signaling pathway, which in turn promotes neuronal survival and growth. Another study also suggested that global deprivation of NGF inactivated the PI3K/Akt signaling pathway and, in contrast, activated mitochondrial apoptosis in cultures of sympathetic neurons *in vitro* [51]. Moreover, it has been reported that the neuronal apoptosis that was induced by NGF deprivation in sympathetic neurons resulted in massive apoptotic cell death [52]. All of these studies suggest that NGF suppression in the nervous system induces apoptosis through inhibition of the PI3K/Akt signaling.

To verify the neurotoxic effects of HD on NGF, the expression level of NGF was tested in the rat sciatic nerve that was exposed to HD. The expression level of NGF in the rat group exposed to HD was lower than that of the control group. To verify the neurotoxicity of HD *in vitro*, the expression level of the NGF protein in HD-exposed VSC4.1 cells was tested. The expression of NGF was significantly lower in the cells exposed to HD than in the control group. Additionally, NGF supplementation reversed the apoptotic effect associated with the decreased expression of p-Akt and p-Bad, and the increased activation of mitochondrial apoptosis observed in HD-exposed VSC4.1 cells. However, LY294002 blocked the protective effects of NGF in VSC4.1 cells that were exposed to HD. Taken together, our results indicate that HD inhibited the NGF expression and suppressed NGF, which subsequently induced neuronal apoptosis via the PI3K/Akt signaling pathway in the rat sciatic nerve.

In conclusion, our study showed that HD exposure suppresses the phosphorylation levels of Akt and Bad, increases the dimerization of Bad and Bcl-xL in the mitochondria, leads to the release of cytochrome *c* from the mitochondria and the activation of caspase-3, and finally induces apoptosis in VSC4.1 cells *in vitro* and the rat sciatic nerve *in vivo*. The toxic effects of HD were eliminated by the co-administration of IGF-1. Moreover, HD exposure also inhibited NGF expression. On the other hand, NGF supplementation reversed the decreased expression levels of p-Akt and p-Bad, and also reversed the increased activation of mitochondrial apoptotic pathway in HD-exposed VSC4.1 cells. However, the protective effects of NGF were blocked by LY294002 administration in the VSC4.1 cells exposed to HD. These results suggest that HD induces apoptosis in rat sciatic nerve by inhibiting NGF and downstream PI3K/Akt signaling. Our findings may provide a novel clue for further clarification of the mechanisms of HD-induced neurotoxicity. Further studies are still needed to demonstrate the molecular mechanism of HD-suppressed expression of NGF in the sciatic nerve of rats exposed to HD.

## Abbreviations

HD: 2,5-hexanedione
PI3K: phosphatidylinositol3-kinase
MMP: mitochondrial membrane potential
NGF: nerve growth factor

## References

[1] Sun Y., Lin Y., Li H., Liu J., Sheng X. and Zhang W. (2012) 2,5-Hexanedione induces human ovarian granulosa cell apoptosis through BCL-2, BAX, and CASPASE-3 signaling pathways. Arch. Toxicol. 86, 205–215 10.1007/s00204-011-0745-7 21901545

[2] Yin H., Guo Y., Zeng T., Zhao X. and Xie K. (2013) Correlation between Levels of 2, 5-hexanedione and pyrrole adducts in tissues of rats exposure to n-hexane for 5-days. PLoS One 8, e76011 10.1371/journal.pone.0076011 24098756 PMC3786887

[3] Yasmen N., Aziz M.A., Tajmim A., Akter I., Hazra A.K. and Rahman S.M.M. (2018) Analgesic and anti-inflammatory activities of diethyl ether and n-hexane extract of polyalthia suberosa leaves. Evidence Based Complement. Altern. Med. 2018, 5617234 10.1155/2018/5617234 PMC5827878 29599807

[4] Lehning E.J., Jortner B.S., Fox J.H., Arezzo J.C., Kitano T. and LoPachin R.M. (2000) gamma-Diketone peripheral neuropathy - I. Quantitative morphometric analyses of axonal atrophy and swelling. Toxicol. Appl. Pharmacol. 165, 127–140 10.1006/taap.2000.8937 10828208

[5] LoPachin R.M., Jortner B.S., Reid M.L. and Das S. (2003) gamma-Diketone central neuropathy: quantitative morphometric analysis of axons in rat spinal cord white matter regions and nerve roots. Toxicol. Appl. Pharmacol. 193, 29–46 10.1016/j.taap.2003.07.005 14613714

[6] Bian X., Jin W., Gu Q., Zhou X., Xi Y., Tu R. et al. (2018) Subcritical n-hexane/isopropanol extraction of lipid from wet microalgal pastes of Scenedesmus obliquus. World J. Microbiol. Biotechnol. 34, 39 10.1007/s11274-018-2421-z 29460187

[7] Spencer P.S. and Schaumburg H.H. (1975) Experimental neuropathy produced by 2,5-hexanedione - major metabolite of neurotoxic industrial solvent methyl normal-butyl ketone. J. Neurol. Neurosurg. Psychiatry 38, 771–775 10.1136/jnnp.38.8.771 171344 PMC492070

[8] Guan H., Piao H., Qian Z., Zhou X., Sun Y., Gao C. et al. (2017) 2,5-Hexanedione induces autophagic death of VSC4.1 cells via a PI3K/Akt/mTOR pathway. Mol. Biosyst. 13, 1993–2005 10.1039/C7MB00001D 28752163

[9] Ogawa Y., Shimizu H. and Kim S.U. (1996) 2,5-Hexanedione induced apoptosis in cultured mouse DRG neurons. Int. Arch. Occup. Environ. Health 68, 495–497 10.1007/BF00377875 8891791

[10] Strange P., Moller A., Ladefoged O., Lam H.R., Larsen J.J. and Arliensoborg P. (1991) Total number and mean cell-volume of neocortical neurons in rats exposed to 2,5-hexanedione with and without acetone. Neurotoxicol. Teratol. 13, 401–406 10.1016/0892-0362(91)90088-E 1921919

[11] Torres M.E., dos Santos A.P.M., Goncalues L.L., Andrade V., Batoreu M.C. and Mateus M.L. (2014) Role of N-acetylcysteine in protecting against 2,5-hexanedione neurotoxicity in a rat model: changes in urinary pyrroles levels and motor activity performance. Environ. Toxicol. Pharmacol. 38, 807–813 10.1016/j.etap.2014.09.008 25305742

[12] Schorey J.S. and Cooper A.M. (2003) Macrophage signalling upon mycobacterial infection: the MAP kinases lead the way. Cell. Microbiol. 5, 133–142 10.1046/j.1462-5822.2003.00263.x 12614457

[13] Cardone M.H., Roy N., Stennicke H.R., Salvesen G.S., Franke T.F., Stanbridge E. et al. (1998) Regulation of cell death protease caspase-9 by phosphorylation. Science 282, 1318–1321 10.1126/science.282.5392.1318 9812896

[14] Datta S.R., Dudek H., Tao X., Masters S., Fu H.A., Gotoh Y. et al. (1997) Akt phosphorylation of BAD couples survival signals to the cell-intrinsic death machinery. Cell 91, 231–241 10.1016/S0092-8674(00)80405-5 9346240

[15] Chen K., Wang N., Diao Y., Dong W., Sun Y., Liu L. et al. (2017) Hydrogen-rich saline attenuates brain injury induced by cardiopulmonary bypass and inhibits microvascular endothelial cell apoptosis via the PI3K/Akt/GSK3β signaling pathway in rats. Cell. Physiol. Biochem. 43, 1634–1647 10.1159/000484024 29040978

[16] Vara JAF, Casado E., de Castro J., Cejas P., Belda-Iniesta C. and Gonzalez-Baron M. (2004) PI3K/Akt signalling pathway and cancer. Cancer Treat. Rev. 30, 193–204 10.1016/j.ctrv.2003.07.007 15023437

[17] Li Y., Jiang Y., Wan Y., Zhang L., Tang W., Ma J. et al. (2013) Medroxyprogestogen enhances apoptosis of SKOV-3 cells via inhibition of the PI3K/Akt signaling pathway. J. Biomed. Res. 27, 43–50 23554793 10.7555/JBR.27.20120051 PMC3596754

[18] Zhou J. and Jia Y. (2017) TRPC channels and programmed cell death. In Transient Receptor Potential Canonical Channels and Brain Diseases, vol. 976 (Wang Y., ed.), pp. 47–60, Springer Nature, U.S.A. 10.1007/978-94-024-1088-4_5 28508312

[19] Blume-Jensen P., Janknecht R. and Hunter T. (1998) The Kit receptor promotes cell survival via activation of PI 3-kinase and subsequent Akt-mediated phosphorylation of Bad on Ser136. Curr. Biol. 8, 779–782 10.1016/S0960-9822(98)70302-1 9651683

[20] Datta S.R., Katsov A., Hu L., Petros A., Fesik S.W., Yaffe M.B. et al. (2000) 14-3-3 proteins and survival kinases cooperate to inactivate BAD by BH3 domain phosphorylation. Mol. Cell 6, 41–51 10.1016/S1097-2765(05)00012-2 10949026

[21] Liu L.-T., Liang L., Wang W., Yan C.-Q., Zhang J., Xiao Y.-C. et al. (2018) Isolariciresinol-9-O–L-arabinofuranoside protects against hydrogen peroxide-induced apoptosis of human umbilical vein endothelial cells via a PI3K/Akt/Bad-dependent pathway. Mol. Med. Rep. 17, 488–494 29115459 10.3892/mmr.2017.7865

[22] Hsu S.Y., Kaipia A., Zhu L. and Hsueh A.J.W. (1997) Interference of BAD (Bcl-xL/Bcl-2-associated death promoter)-induced apoptosis in mammalian cells by 14-3-3 isoforms and P11. Mol. Endocrinol. 11, 1858–1867 9369453 10.1210/mend.11.12.0023

[23] Li Y., Zeng M., Chen W., Liu C., Wang F., Han X. et al. (2014) Dexmedetomidine reduces isoflurane-induced neuroapoptosis partly by preserving PI3K/Akt pathway in the hippocampus of neonatal rats. PLoS One 9, e93639 24743508 10.1371/journal.pone.0093639 PMC3990549

[24] Blum R. and Konnerth A. (2005) Neurotrophin-mediated rapid signaling in the central nervous system: mechanisms and functions. Physiology 20, 70–78 10.1152/physiol.00042.2004 15653842

[25] Keefe K.M., Sheikh I.S. and Smith G.M. (2017) Targeting neurotrophins to specific populations of neurons: NGF, BDNF, and NT-3 and their relevance for treatment of spinal cord injury. Int. J. Mol. Sci. 18, 548 10.3390/ijms18030548 28273811 PMC5372564

[26] Tabakman R., Lecht S., Sephanova S., Arien-Zakay H. and Lazarovici P. (2004) Interactions between the cells of the immune and nervous system: neurotrophins as neuroprotection mediators in CNS injury. In Ngf and Related Molecules in Health and Disease, vol. 146 (Aloe L. and Calza L., eds), pp. 387–401, Elsevier, Amsterdam 10.1016/s0079-6123(03)46024-x 14699975

[27] Pandini G., Satriano C., Pietropaolo A., Giani F., Travaglia A., La Mendola D. et al. (2016) The inorganic side of NGF: Copper(II) and Zinc(II) affect the NGF mimicking signaling of the N-terminus peptides encompassing the recognition domain of TrkA receptor. Front. Neurosci. 10, 569 10.3389/fnins.2016.00569 28090201 PMC5201159

[28] Shimoke K., Amano H., Kishi S., Uchida H., Kudo M. and Ikeuchi T. (2004) Nerve growth factor attenuates endoplasmic reticulum stress-mediated apoptosis via suppression of caspase-12 activity. J. Biochem. (Tokyo) 135, 439–446 10.1093/jb/mvh053 15113843

[29] Shimoke K., Kishi S., Utsumi T., Shimamura Y., Sasaya H., Oikawa T. et al. (2005) NGF-induced phosphatidylinositol 3-kinase signaling pathway prevents thapsigargin-triggered ER stress-mediated apoptosis in PC12 cells. Neurosci. Lett. 389, 124–128 10.1016/j.neulet.2005.07.030 16095815

[30] Fiore M., Mancinelli R., Aloe L., Laviola G., Sornelli F., Vitali M. et al. (2009) Hepatocyte growth factor, vascular endothelial growth factor, glial cell-derived neurotrophic factor and nerve growth factor are differentially affected by early chronic ethanol or red wine intake. Toxicol. Lett. 188, 208–213 10.1016/j.toxlet.2009.04.013 19397965

[31] Cavaletti G., Pezzoni G., Pisano C., Oggioni N., Sala F., Zoia C. et al. (2002) Cisplatin-induced peripheral neurotoxicity in rats reduces the circulating levels of nerve growth factor. Neurosci. Lett. 322, 103–106, (art. Pii s0304-3940(02)00091-5) 10.1016/S0304-3940(02)00091-5 11958854

[32] Namgung U. and Xia Z.G. (2000) Arsenite-induced apoptosis in cortical neurons is mediated by c-Jun N-terminal protein kinase 3 and p38 mitogen-activated protein kinase. J. Neurosci. 20, 6442–6451 10.1523/JNEUROSCI.20-17-06442.2000 10964950 PMC6772983

[33] Li S., Guan H., Qian Z., Sun Y., Gao C., Li G. et al. (2017) Taurine inhibits 2,5-hexanedione-induced oxidative stress and mitochondria-dependent apoptosis in PC12 cells. Ind. Health 55, 108–118 10.2486/indhealth.2016-0044 27840369 PMC5383408

[34] Wang Q.-S., Song F., Zhao X., L-y Hou and Xie K.-Q. (2007) Expression changes of apoptotic-related proteins in nerve tissues of rats treated with allyl chloride. Toxicology 231, 58–67 10.1016/j.tox.2006.11.071 17194518

[35] Gilbert S.G. and Maurissen JPJ (1982) Assessment of the effects of acrylamide, methylmercury, and 2,5-hexanedione on motor functions in mice. J. Toxicol. Environ. Health 10, 31–41 10.1080/15287398209530228 7131587

[36] Smith R.G., Alexianu M.E., Crawford G., Nyormoi O., Stefani E. and Appel S.H. (1994) Cytotoxicity of immunoglobulins from amyotrophic-lateral-sclerosis patients on a hybrid motoneuron cell-line. PNAS 91, 3393–3397 10.1073/pnas.91.8.3393 8159758 PMC43583

[37] Hao J., Li S., Shi X., Qian Z., Sun Y., Wang D. et al. (2018) Bone marrow mesenchymal stem cells protect against n-hexane-induced neuropathy through beclin 1-independent inhibition of autophagy. Sci. Rep. 8, 4516 10.1038/s41598-018-22857-x 29540747 PMC5852116

[38] Kishi S., Shimoke K., Nakatani Y., Shimada T., Okumura N., Nagai K. et al. (2010) Nerve growth factor attenuates 2-deoxy-D-glucose-triggered endoplasmic reticulum stress-mediated apoptosis via enhanced expression of GRP78. Neurosci. Res. 66, 14–21 10.1016/j.neures.2009.09.003 19766678

[39] Guo X.-q., Cao Y.-l., Hao F., Yan Z.-r., Wang M.-l. and Liu X.-w. (2017) Tangeretin alters neuronal apoptosis and ameliorates the severity of seizures in experimental epilepsy-induced rats by modulating apoptotic protein expressions, regulating matrix metalloproteinases, and activating the PI3K/Akt cell survival pathway. Adv. Med. Sci. 62, 246–253 10.1016/j.advms.2016.11.011 28501723

[40] Maddika S., Ande S.R., Panigrahi S., Paranjothy T., Weglarczyk K., Zuse A. et al. (2007) Cell survival, cell death and cell cycle pathways are interconnected: implications for cancer therapy. Drug Resist. Updat. 10, 13–29 10.1016/j.drup.2007.01.003 17303468

[41] Li H., Tang Z., Chu P., Song Y., Yang Y., Sun B. et al. (2018) Neuroprotective effect of phosphocreatine on oxidative stress and mitochondrial dysfunction induced apoptosis in vitro and in vivo: involvement of dual PI3K/Akt and Nrf2/HO-1 pathways. Free Radic. Biol. Med. 120, 228–238 10.1016/j.freeradbiomed.2018.03.014 29559323

[42] Datta S.R., Ranger A.M., Lin M.Z., Sturgill J.F., Ma Y.C., Cowan C.W. et al. (2002) Survival factor-mediated BAD phosphorylation raises the mitochondrial threshold for apoptosis. Dev. Cell 3, 631–643 10.1016/S1534-5807(02)00326-X 12431371

[43] Nguyen K.T., Zong C.S., Uttamsingh S., Sachdev P., Bhanot M., Le M.T. et al. (2002) The role of phosphatidylinositol 3-kinase, Rho family GTPases, and STAT3 in Ros-induced cell transformation. J. Biol. Chem. 277, 11107–11115 10.1074/jbc.M108166200 11799110

[44] Tao S.-C., Yuan T., Rui B.-Y., Zhu Z.-Z., Guo S.-C. and Zhang C.-Q. (2017) Exosomes derived from human platelet-rich plasma prevent apoptosis induced by glucocorticoid-associated endoplasmic reticulum stress in rat osteonecrosis of the femoral head via the Akt/Bad/Bcl-2 signal pathway. Theranostics 7, 733–750 10.7150/thno.17450 28255363 PMC5327646

[45] Jung S.-Y., Kim D.-Y., Yune T.Y., Shin D.-H., Baek S.-B. and Kim C.-J. (2014) Treadmill exercise reduces spinal cord injury-induced apoptosis by activating the PI3K/Akt pathway in rats. Exp. Therapeutic Med. 7, 587–593 10.3892/etm.2013.1451 PMC3919853 24520250

[46] Sloniecka M., Backman L.J. and Danielson P. (2016) Antiapoptotic Effect of Acetylcholine in Fas-Induced Apoptosis in Human Keratocytes. Investigative Ophthalmol. Visual Sci. 57, 5892–5902 10.1167/iovs.16-19707 27802519

[47] Kristiansen M. and Ham J. (2014) Programmed cell death during neuronal development: the sympathetic neuron model. Cell Death Differ. 21, 1025–1035 10.1038/cdd.2014.47 24769728 PMC4207485

[48] Deshmukh M., Vasilakos J., Deckwerth T.L., Lampe P.A. and Johnson E.M. (1996) Genetic and metabolic status of NGF-deprived sympathetic neurons saved by an inhibitor of ICE family proteases. J. Cell Biol. 135, 1341–1354 10.1083/jcb.135.5.1341 8947555 PMC2121082

[49] Wang Z.M., Zhong C.Y. and Zhao G.J. (2017) Polyphenol epigallocatechin-3-gallate alleviates high Nucose high induced H9C2 cell damage through PI3K/Akt pathway. Eur. Rev. Med. Pharmacol. Sci. 21, 4236–4242 29028073

[50] Yang E., Zha J.P., Jockel J., Boise L.H., Thompson C.B. and Korsmeyer S.J. (1995) Bad, A Heterodimeric Partner For BCL-X(L) and BCL-2, displaces bax and promotes cell-death. Cell 80, 285–291 10.1016/0092-8674(95)90411-5 7834748

[51] Zhang H., Wu F., Kong X., Yang J., Chen H., Deng L. et al. (2014) Nerve growth factor improves functional recovery by inhibiting endoplasmic reticulum stress-induced neuronal apoptosis in rats with spinal cord injury. J. Transl. Med. 12, (art. 130) 10.1186/1479-5876-12-130 PMC4039547 24884850

[52] Zilz T.R., Griffiths H.R. and Coleman M.D. (2007) Apoptotic and necrotic effects of hexanedione derivatives on the human neuroblastoma line SK-N-SH. Toxicology 231, 210–214 10.1016/j.tox.2006.12.002 17229511

